# Progress of American Surgery in Europe

**Published:** 1859-03

**Authors:** 


PROGRESS OF AMERICAN SURGERY IN EUROPE.—It must be highly gratifying to the friends of humanity and of scientific surgery everywhere, to find that the operation for the cure of that loathsome and disgusting complaint, vesico-vaginal fistule, so greatly improved by our countrymen, Drs. Sims and Bozeman, as to entitle them to the gratitude of the whole medical profession, is at present attracting so much attention both in the United States and in Europe. There is hardly a number of any of our foreign exchanges, in the different languages of Europe, that does not contain either an essay upon the subject or an account of some case illustrative of a new triumph. Dr. Simpson, as usual, takes the lead, and in a late number of the *London Medical Times and Gazette,* he has quite an elaborate paper on the treatment of this affection, in which he proposes an improvement upon Dr. Bozeman's method, consisting in the substitution of a wire splint for the metallic button. Dr. Baker Brown has also published an able brochure upon the subject, detailing the successful treatment of eleven cases.

During his recent visit to Europe, Dr. Bozeman received the kindest attention from his professional brethren, and was invited to exhibit his operation in different hospitals, upon the living subject. In London, a case was placed under his charge by Mr. Erichsen, in University College Hospital; at Edinburgh, he operated at the Royal Infirmary of that city, and also in the private practice of Professor Simpson; the doors of the Royal Infirmary of Glasgow were likewise thrown open to him; and at Paris he operated for M. Robert, at the Hôtel-Dieu. Of these cases, three were perfectly successful; in one, the parts reopened at the end of the sixth day; and in another, death occurred from peritonitis. It is proper to add that none of these cases had been properly prepared for the operation, and that Dr. Bozeman had no agency whatever in the the after-treatment in any. The woman operated upon in the Royal Infirmary at Edinburgh was a peculiarly bad subject, having been an intemperate, dissolute creature.

